# Supplementary material for: Lionheart LincRNA alleviates cardiac systolic dysfunction under pressure overload
Source: Commun Biol. 2020 Aug 13;3:434. doi: 10.1038/s42003-020-01164-0 (PMC7426859; doi:10.1038/s42003-020-01164-0)
Supplement: Supplementary file 2 — Description of Additional Supplementary Files [file 42003_2020_1164_MOESM2_ESM.pdf]

# Description of Additional Supplementary Files

**File Name**: **Supplementary Data 1**
**Description** the source data file
