# Supplementary material for: Lionheart LincRNA alleviates cardiac systolic dysfunction under pressure overload
Source: Commun Biol. 2020 Aug 13;3:434. doi: 10.1038/s42003-020-01164-0 (PMC7426859; doi:10.1038/s42003-020-01164-0)
Supplement: Supplementary file 3 — Reporting Summary [file 42003_2020_1164_MOESM3_ESM.pdf]

nature research

Corresponding author(s): Koh Ono

Last updated by author(s): Jul 2, 2020

# Reporting Summary

Nature Research wishes to improve the reproducibility of the work that we publish. This form provides structure for consistency and transparency in reporting. For further information on Nature Research policies, see Authors & Referees and the Editorial Policy Checklist.

## Statistics

For all statistical analyses, confirm that the following items are present in the figure legend, table legend, main text, or Methods section.

| n/a | Confirmed | |
|---|---|---|
| ☐ | ☒ | The exact sample size (*n*) for each experimental group/condition, given as a discrete number and unit of measurement |
| ☐ | ☒ | A statement on whether measurements were taken from distinct samples or whether the same sample was measured repeatedly |
| ☐ | ☒ | The statistical test(s) used AND whether they are one- or two-sided<br>*Only common tests should be described solely by name; describe more complex techniques in the Methods section.* |
| ☒ | ☐ | A description of all covariates tested |
| ☐ | ☒ | A description of any assumptions or corrections, such as tests of normality and adjustment for multiple comparisons |
| ☐ | ☒ | A full description of the statistical parameters including central tendency (e.g. means) or other basic estimates (e.g. regression coefficient) AND variation (e.g. standard deviation) or associated estimates of uncertainty (e.g. confidence intervals) |
| ☐ | ☒ | For null hypothesis testing, the test statistic (e.g. *F*, *t*, *r*) with confidence intervals, effect sizes, degrees of freedom and *P* value noted<br>*Give P values as exact values whenever suitable.* |
| ☒ | ☐ | For Bayesian analysis, information on the choice of priors and Markov chain Monte Carlo settings |
| ☒ | ☐ | For hierarchical and complex designs, identification of the appropriate level for tests and full reporting of outcomes |
| ☐ | ☒ | Estimates of effect sizes (e.g. Cohen's *d*, Pearson's *r*), indicating how they were calculated |

*Our web collection on statistics for biologists contains articles on many of the points above.*

## Software and code

Policy information about availability of computer code

| | |
|---|---|
| Data collection | 7900HT Fast Real-Time PCR System (Applied Biosystems), Vevo® 2100 (VISUALSONICS), LAS-3000 (Fujifilm), Amersham Imager 680 (GE Healthcare Life Sciences), ImageJ64 software (NIH), BZ-9000 (Keyence), Scanner 3000 7G (Affymetrix), R ver. 3.5.3 (R Foundation for Statistical Computing), Primer3 (http://primer3.wi.mit.edu), DAVID bioinformatic resources 6.8 (https://david.ncifcrf.gov/home.jsp), transmission electron microscopy (H-7650, Hitachi), mass spectrometer (TripleTOF 5600+ system, SCIEX), ProteinPilot software version 4.5beta (SCIEX), Progenesis QI for Poteomics software (Nonlinear Dynamics). |
| Data analysis | Data were analyzed by GraphPad Prism 7.0. |

For manuscripts utilizing custom algorithms or software that are central to the research but not yet described in published literature, software must be made available to editors/reviewers. We strongly encourage code deposition in a community repository (e.g. GitHub). See the Nature Research guidelines for submitting code & software for further information.

## Data

Policy information about availability of data

All manuscripts must include a data availability statement. This statement should provide the following information, where applicable:
- Accession codes, unique identifiers, or web links for publicly available datasets
- A list of figures that have associated raw data
- A description of any restrictions on data availability

The datasets generated during and/or analyzed during the current study are included in this published article and are available from the corresponding author on reasonable request.

# Field-specific reporting

Please select the one below that is the best fit for your research. If you are not sure, read the appropriate sections before making your selection.

☒ Life sciences ☐ Behavioural & social sciences ☐ Ecological, evolutionary & environmental sciences

For a reference copy of the document with all sections, see nature.com/documents/nr-reporting-summary-flat.pdf

# Life sciences study design

All studies must disclose on these points even when the disclosure is negative.

| | |
|---|---|
| Sample size | Sample sizes were determined by power calculations based on effect sizes previously reported in the literature. |
| Data exclusions | Any mice that experienced complications from surgery were excluded from proceeding with the experiments. This was predetermined criteria before commencing experiments to limit confounding variables. |
| Replication | Experiments were replicated in multiple instances. All attempts at replication were successful. |
| Randomization | Animal allocation to treatment groups (AAV9-Control, AAV9-Lionheart) was randomized. |
| Blinding | All analyses were performed in a uniform and unbiased fashion. |

# Reporting for specific materials, systems and methods

We require information from authors about some types of materials, experimental systems and methods used in many studies. Here, indicate whether each material, system or method listed is relevant to your study. If you are not sure if a list item applies to your research, read the appropriate section before selecting a response.

## Materials & experimental systems

| n/a | Involved in the study | |
|---|---|---|
| ☐ | ☒ | Antibodies |
| ☐ | ☒ | Eukaryotic cell lines |
| ☒ | ☐ | Palaeontology |
| ☐ | ☒ | Animals and other organisms |
| ☐ | ☒ | Human research participants |
| ☒ | ☐ | Clinical data |

## Methods

| n/a | Involved in the study | |
|---|---|---|
| ☒ | ☐ | ChIP-seq |
| ☒ | ☐ | Flow cytometry |
| ☒ | ☐ | MRI-based neuroimaging |

## Antibodies

| | |
|---|---|
| Antibodies used | anti glyceraldehyde-3-phosphate dehydrogenase (GAPDH) (Cell Signaling, 14C10); anti heavy chain cardiac myosin (abcam, BA-G5, ab50967) for MYH6; monoclonal anti-Myosin (Skeletal, Slow) for MYH7 (Sigma-Aldrich, M8421); anti-PURA (Santa Cruz, 80-L, sc-130397); anti-CSRP3 (abcam, ab173301); Anti-rabbit IgG (GE Healthcare); anti-mouse IgG(GE Healthcare) |
| Validation | All antibodies are commercially available and validated by the manufactures for the applications and species used in this study. |

## Eukaryotic cell lines

Policy information about cell lines

| | |
|---|---|
| Cell line source(s) | HEK293T cells. |
| Authentication | Cell lines are authenticated by ATCC. |
| Mycoplasma contamination | We routinely test for mycoplasma contamination, the most recent results showing no contamination in any cell lines used. |
| Commonly misidentified lines (See ICLAC register) | No commonly misidentified lines was used. |

## Animals and other organisms

Policy information about studies involving animals; ARRIVE guidelines recommended for reporting animal research

| | |
|---|---|
| Laboratory animals | All mice used were on a C57BL/6J background. To generate Lionheart knockout mice, C57BL/6J mouse embryonic stem (ES) cells and homologous recombination system were used. |
| Wild animals | C57BL/6J male mice were purchased from Japan SLC. For animal experiments, 8-16 weeks age mice were used. |
| Field-collected samples | Mice were maintained in temperature-controlled rooms with a 14:10 h light:dark cycle in specific pathogen-free conditions at the Institute of Laboratory Animals of Kyoto University Graduate School of Medicine. |
| Ethics oversight | This study was approved by the Kyoto University Ethics Review Board. |

Note that full information on the approval of the study protocol must also be provided in the manuscript.

## Human research participants

Policy information about studies involving human research participants

| | |
|---|---|
| Population characteristics | Patients diagnosed with heart failure by a cardiologist. The number of patients was 58 and all of them were Japanese. Mean age was 56.1, 36 male patients, mean left ventricular ejection fraction was 34.3%. |
| Recruitment | Patients who were hospitalized with a diagnosis of heart failure and were determined to need myocardial biopsy for further examination. All patients provided written informed consent for the procedure and gene expression analyses. |
| Ethics oversight | The Ethics Committees of Osaka Red Cross Hospital and Kyoto University Hospital approved the study protocol. |

Note that full information on the approval of the study protocol must also be provided in the manuscript.
