# Supplementary material for: Lionheart LincRNA alleviates cardiac systolic dysfunction under pressure overload
Source: Commun Biol. 2020 Aug 13;3:434. doi: 10.1038/s42003-020-01164-0 (PMC7426859; doi:10.1038/s42003-020-01164-0)
Supplement: Supplementary file 5 — Supplementary Information [file 42003_2020_1164_MOESM5_ESM.pdf]

Lionheart LincRNA Alleviates Cardiac Systolic Dysfunction under Pressure Overload

Supplementary Information

Supplementary Fig. 1. Vertebrate conservation of continuously upregulated lincRNAs after pressure overload

Supplementary Fig. 2. Sequence of Lionheart and examination if Lionheart is a protein-coding RNA

Supplementary Fig. 3. Genomic locations of mouse *Lionheart* and the orthologues of rat and human

Supplementary Fig. 4. Regulation of *Lionheart*

Supplementary Fig. 5. Site directed mutagenesis of putative CArG box 3 and 5 in -0.6 kb *Lionheart* promoter

Supplementary Fig. 6. Generation of *Lionheart* knockout mouse

Supplementary Fig. 7. Features of *Lionheart*-KO mouse subjected to TAC

Supplementary Fig. 8. Blood Pressure of *Lionheart*-KO mice subjected to TAC

Supplementary Fig. 9. mRNA levels of *Lionheart* neighboring genes in *Lionheart*-KO mouse hearts

Supplementary Fig. 10. Expression changes of lncRNAs in *Lionheart*-KO mouse hearts after TAC surgery

Supplementary Fig. 11. Mitochondrial ultrastructure of *Lionheart*-KO mouse hearts with or without TAC

Supplementary Fig. 12. Protein levels of PURA in *Lionheart*-KO mice

Supplementary Fig. 13. Lionheart levels and PURA protein levels in right ventricles after TAC surgery

Supplementary Fig. 14. Uncropped immunoblot images in main Figures

Supplementary Fig. 15. Uncropped western blot images in Supplementary Figures

Supplementary Table 1. GO-BP term analysis for Lionheart sense-binding protein candidates

Supplementary Table 2. GO-BP term analysis for Lionheart antisense-binding protein candidates

Supplementary Table 3. Patient characteristics in LV biopsy cohort

a

lincRNA-3

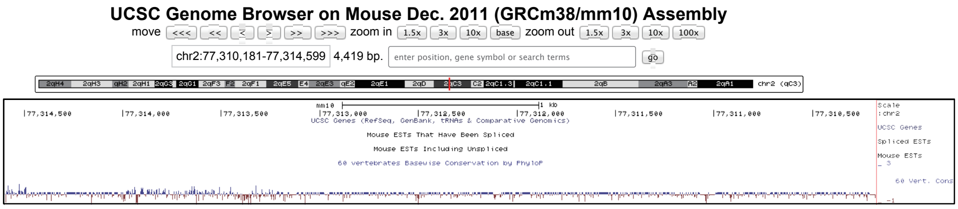

b

lincRNA-5: *Lionheart*

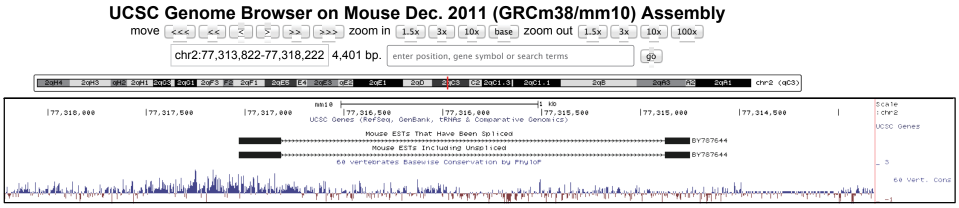

c

lincRNA-7

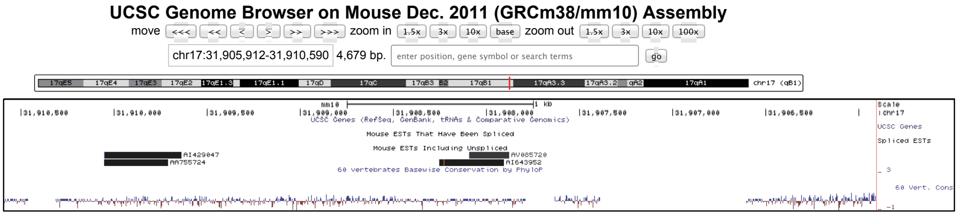

Supplementary Fig. 1. Vertebrate conservation of continuously upregulated lincRNAs after pressure overload. (**a**) lincRNA-3. (**b**) lincRNA-5 (*Lionheart*). (**c**) lincRNA-7.

a

mmu-Lionheart

AAAGTAGGACAAGTAACTGAAGCTTTGTAATCGTTCAGTATGGGGCCTCAACTCTCATCTTAACTCGATGGGATGTGCTTGGGAGAGGCGAG
AAGTGCTTGTAGGAACAATAAATCATGAGAGAGGCATTCTACAGAGTTTATTTTAGCTTTTGCTCCTGACAAGAGAGTGGCATGCCACACTCA
GAGTTCAACGGGTCCTCCGAGCAGAAGTTCTTCTGTCCTTCAAAAGATTTCTCTACTAGTGAGAGAAGAAGAGGGGGCCGATTCCCTGGAAC
TAGTTACTGGTGATTGTGAGCAGCCCACGGATGCTGAAAAACAAACTCATGTCCTCTGGAAGAGCAGCGAGTGCATCTAACCACTGAGCTAT
CTCTCTAGACCCTTAAATAAAAATCTAAAACTC

b

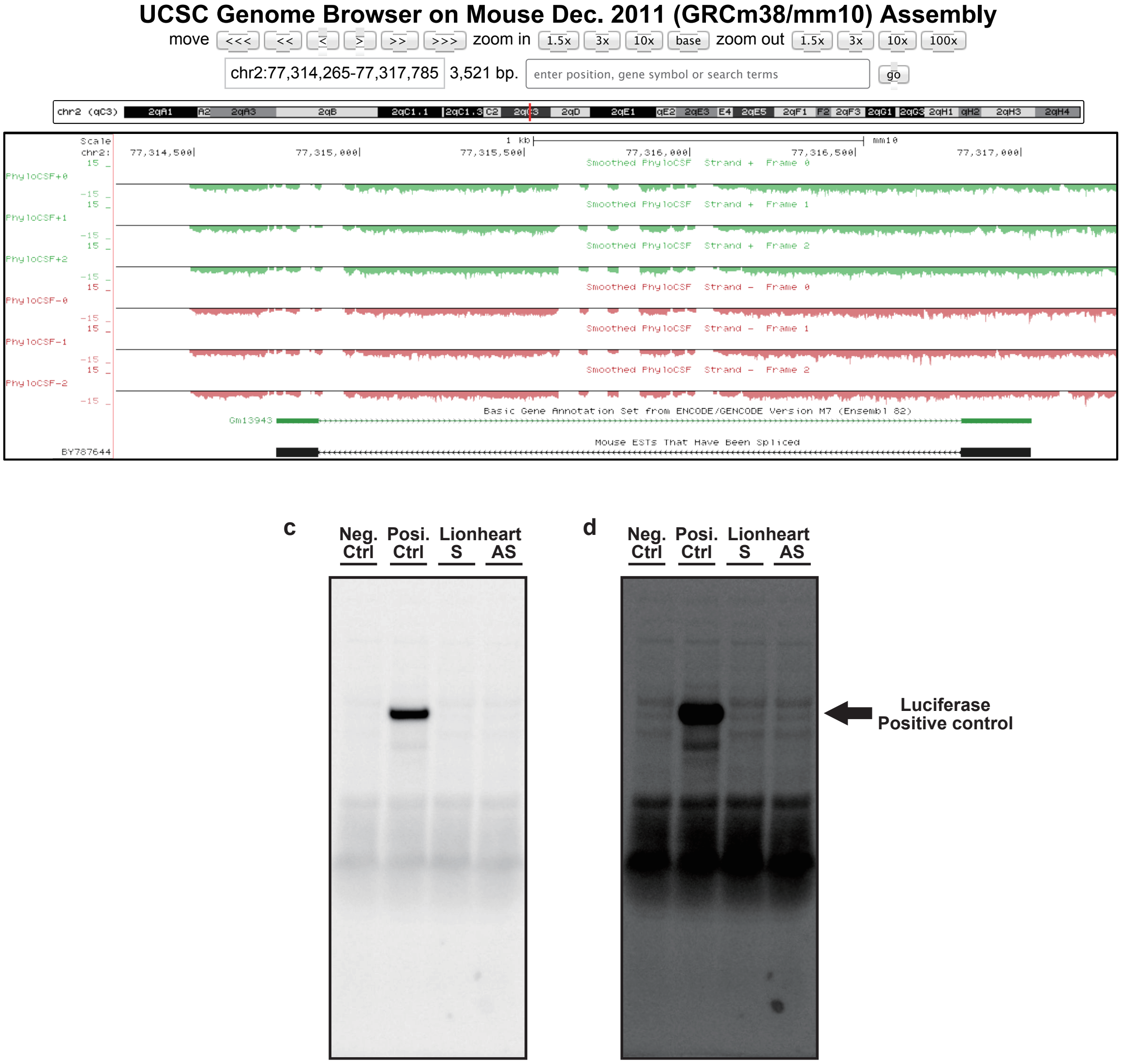

Supplementary Fig. 2. Sequence of Lionheart and examination if Lionheart is a protein-coding RNA. (**a**) Sequence of Lionheart. Sequence in blue is the first exon. Sequence in green is the second exon. (**b**) PhyloCSF bioinformatics analysis indicated Lionheart is a protein-noncoding RNA, because there was no positive signal in exon 1 and 2 of *Lionheart*. (**c** and **d**) In vitro translation assay of Lionheart. (**c**) short exposure. (**d**) long exposure. S: Sense; AS: Antisense.

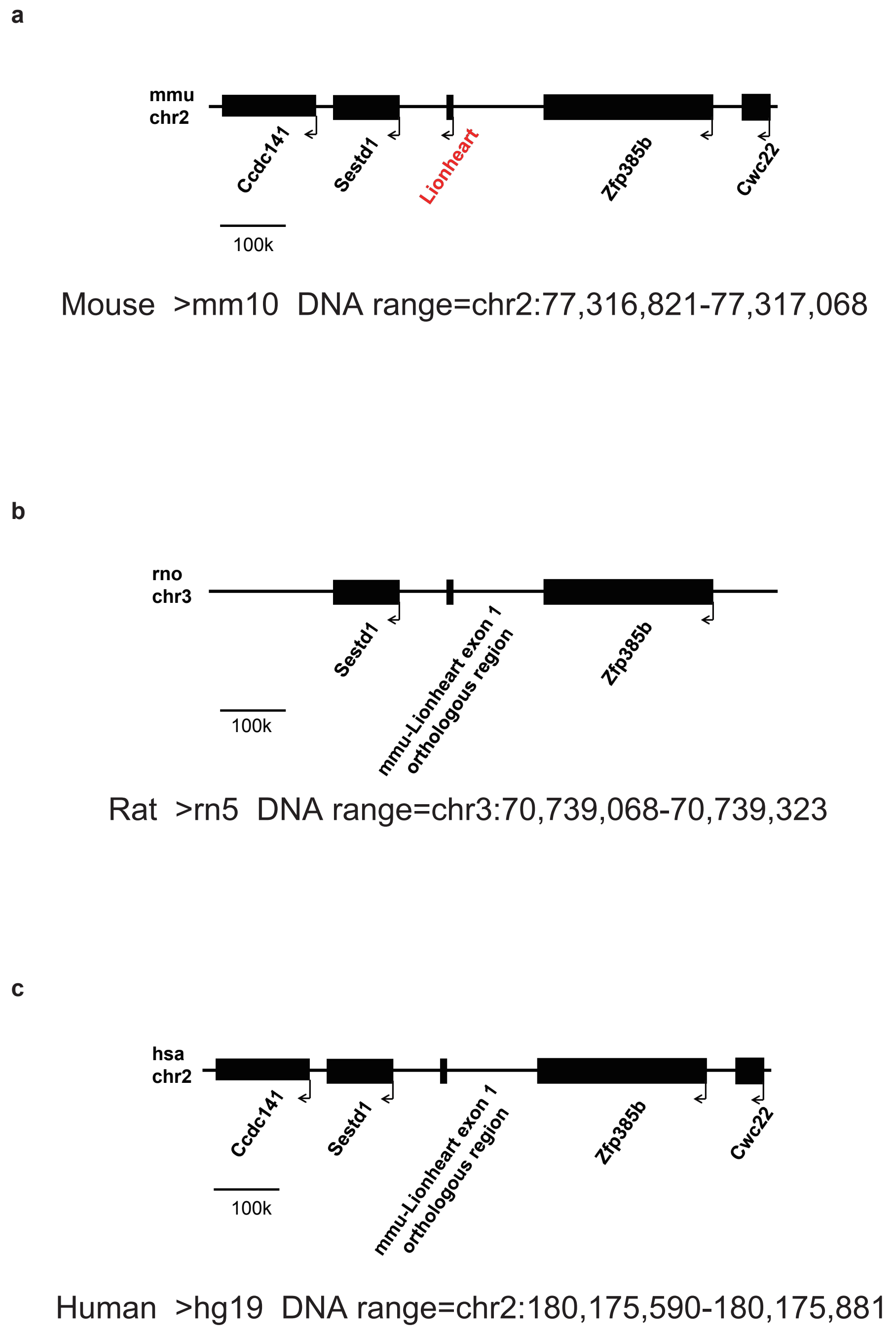

Supplementary Fig. 3. Genomic locations of mouse *Lionheart* and the orthologues of rat and human. (**a**) Genomic location of mouse *Lionheart* and the neighbring genes. DNA range indicates the position of *Lionheart* first exon. (**b**) Genomic location of rat orthologue of *Lionheart* first exon and the the neighbring genes. (**c**) Genomic location of human orthologue of *Lionheart* first exon and the the neighbring genes. DNA ranges indicate the position of the orthologous regions of *Lionheart* first exon. Ccdc141: Coiled-coil domain containing 141; Sestd1: SEC14 and spectrin domains 1; Zfp385b: Zinc finger protein 385B; Cwc22: CWC22 Spliceosome-associated protein homolog.

a

b

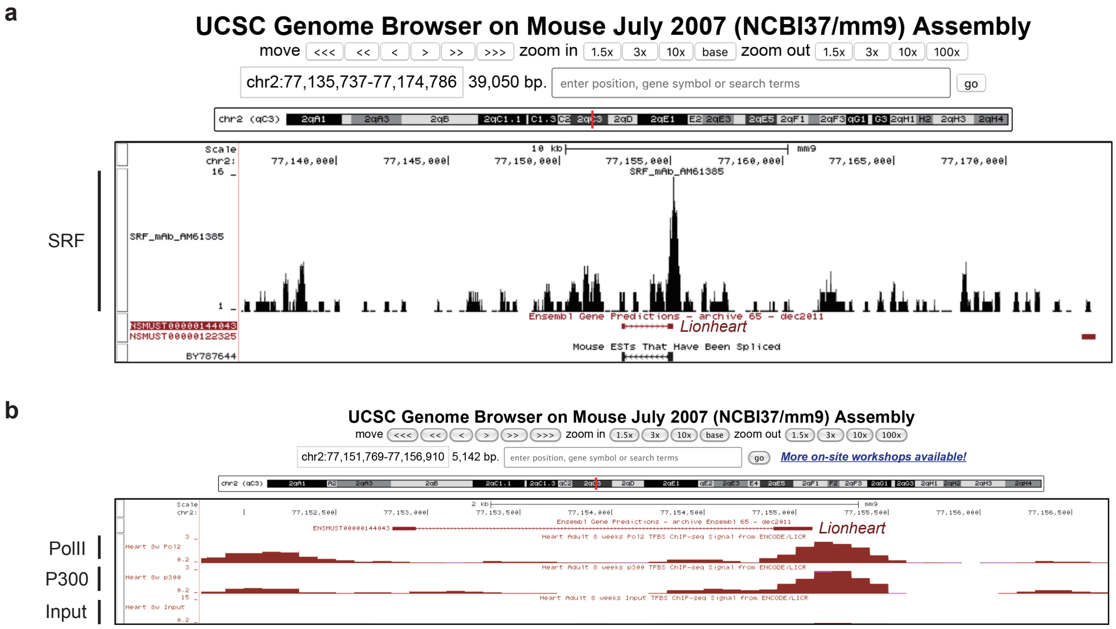

c

Lionheart promoter -0.6 k

AATTGACTTATGAGTTACACACTGTATTCAATTAACTTACGTATTATTATGCCAGTACT
CAATTTATTTTACTTATGATACCCATGAAATGACCTGAGGGCAAATATATTAAAATATT
TATAACTCTATTTTTAAACTCTGACCAGTCTATTTTATTCATGTATTTTACAGCTGCAG
ATTTCTTTGTAGAGTGAGTCTGTCTTTGAAAAGCAATGCCCCAAACAGCGATGCGTTGC
TTTCTGTCTAACTGGTATTTATGTCAAAGCCCCATCAAATCCATTGTTTCATTTGAATC
CTGAATTGTACTATTATGACATCATTCAGATTAGCCACATTCAGCTTCTCAGCCTCCTC
AGATTGAAAACCAGAGGATTTTCAAAATGAACTTGACTTTGCTTCACTGAAATTGTTCT
GCAATGCTCCTGAGGAGACAGCTGTCAACTCCGTTGGCTACAGCCCCAGTGCTGGCTTG
CGCTTACAGACAGGGTTCTGCAGTCAGGTCAGAGCCGTTAGCCTCATGCTAACTATGGT
TGCAGCTGTCTTTGGTCATGCTGAGCATGGTCCAGCACCGTCATCTATCAGAGCACTGT
GCTGTGCCTGCCTTAAAAGCAGATCCCTGTAAAGTAGGACAAGTAACTGAAGCTTTGTA

1 2 3 4 5 6 *Lionheart*

Supplementary Fig. 4. Regulation of *Lionheart*. (**a**) ChIP-seq data using an anti-SRF antibody in the *Lionheart* locus. (**b**) ChIP-seq data using anti-PolII and anti-P300 antibodies in the *Lionheart* locus. (**c**) -0.6 kb promoter sequence and transcriptional start site of *Lionheart*. Rectangles in green are possible CArG sites in the *Lionheart* promoter.

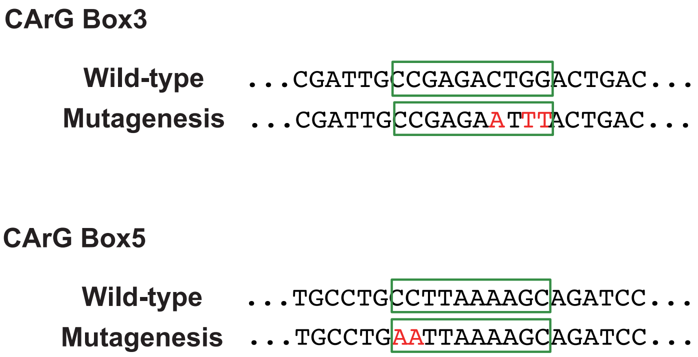

Supplementary Fig. 5. Site directed mutagenesis of putative CArG box 3 and 5 in -0.6 kb *Lionheart* promoter. Nucleotides in red represent mutated nucleotides.

a

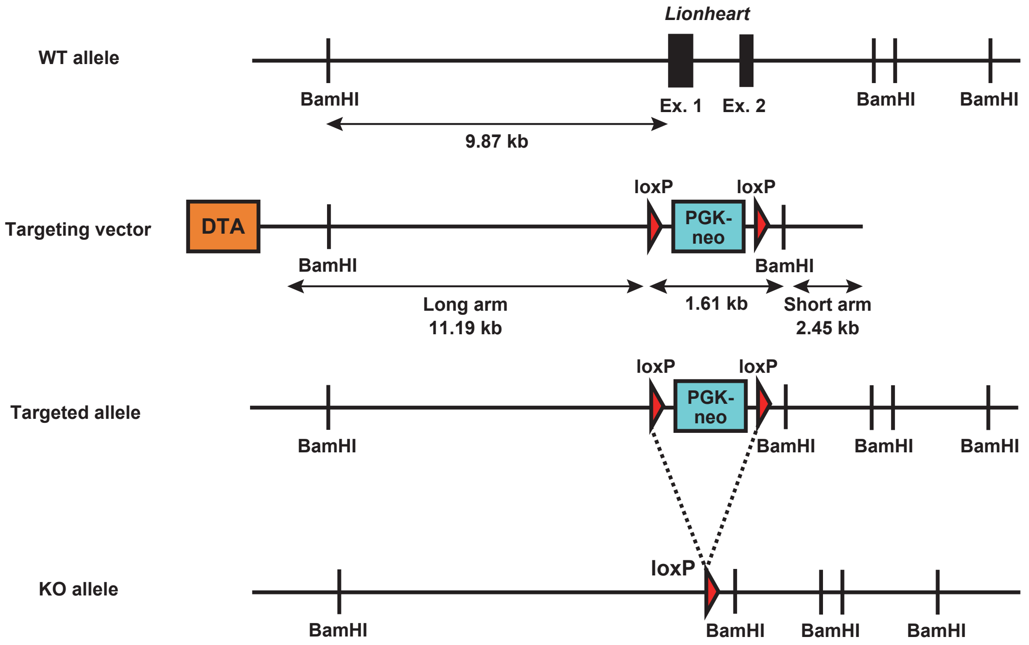

b

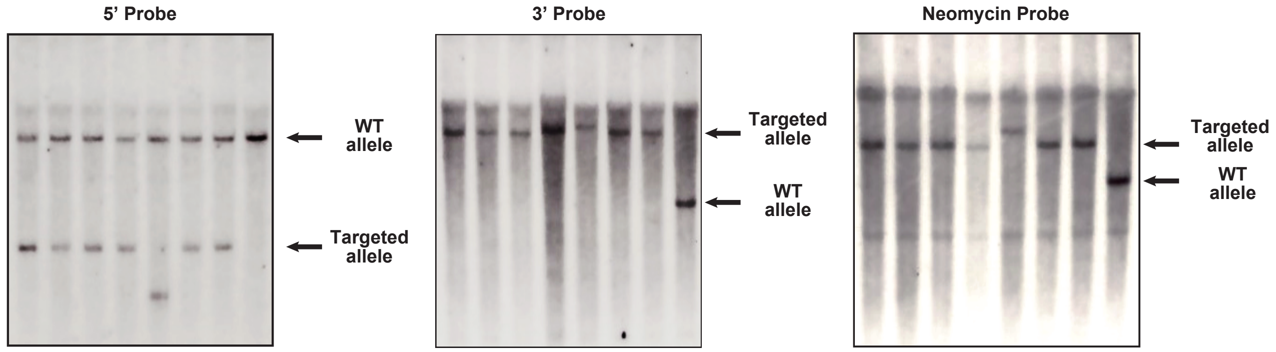

Supplementary Fig. 6. Generation of *Lionheart* knockout mouse. (**a**) Strategy for generation of *Lionheart* deleted mouse. *Lionheart* locus including exon1 and exon2 was replaced by PGK-neomycin sequence flanked by loxP. WT: Wild-type; KO: Knockout. (**b**) Southern blotting data to confirm homologous recombination in the *Lionheart* locus.

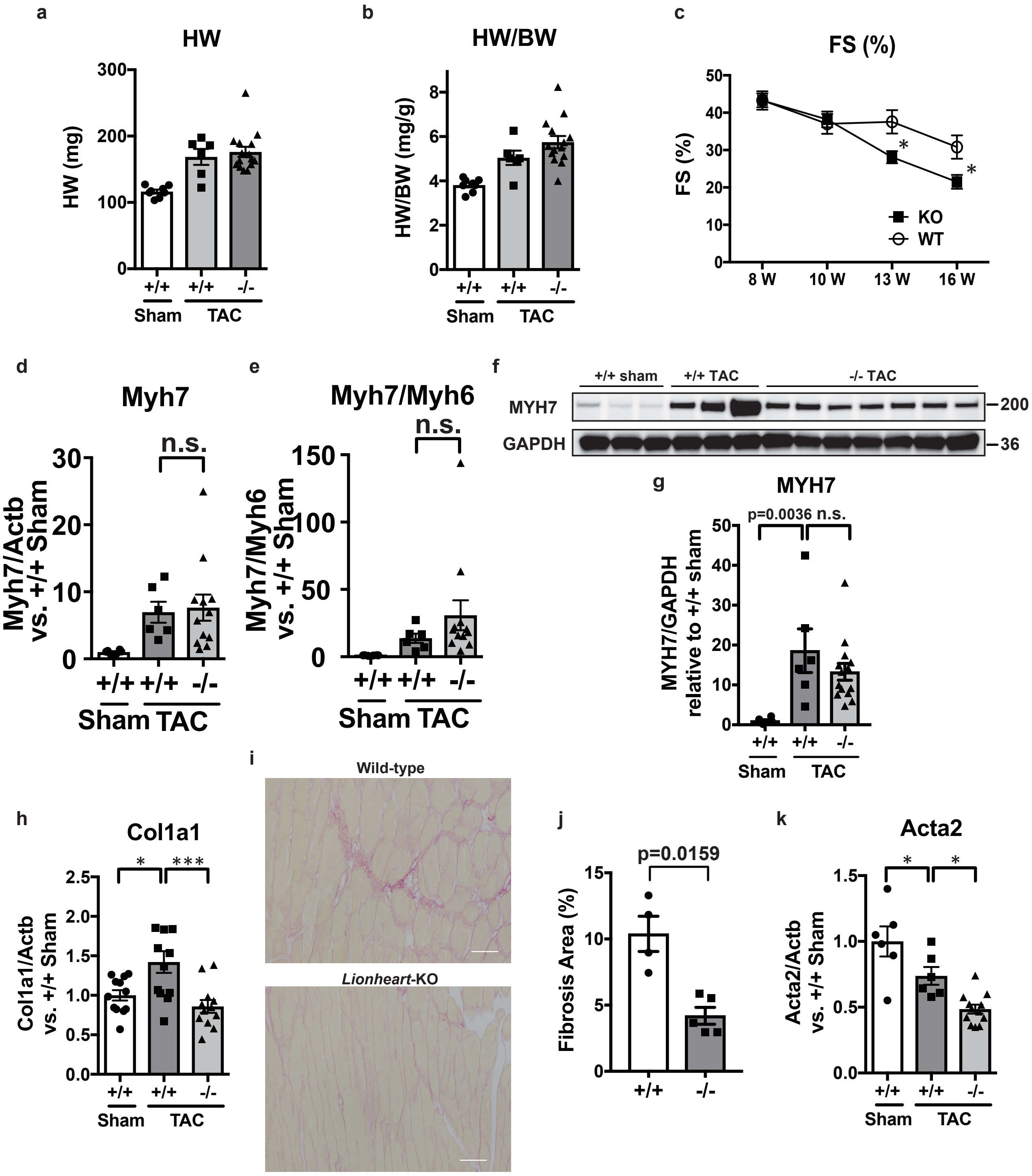

Supplementary Fig. 7. Features of *Lionheart*-KO mouse subjected to TAC. (**a** and **b**) Heart weight (HW) (**a**) and ratio of HW to body weight (BW) (**b**) at 8 weeks after TAC (16 weeks of age). (**c**) Fractional shortening (FS) changes assessed by echocardiography after TAC. WT: n = 8−12; KO: n = 12−17. *, compared with WT at same age. (**d** and **e**) mRNA levels of *Myh7* (**d**) *and ratio of* Myh7 *to* Myh6 (**e**). (**f** and **g**) Representative image of western blotting for MYH7 (**f**) and the quantification (**g**). (**h**) mRNA levels of *Col1a1* by qPCR. n = 12. (**i**) Picrosirius red staining of WT and *Lionheart*-KO mouse at 8 weeks after TAC. Scale bar = 40 μm. (**j**) Quantification of the fibrosis area at 8 weeks after TAC. n = 4−5. (**k**) mRNA levels of *Acta2*. *, $p < 0.05$; ***, $p < 0.001$.

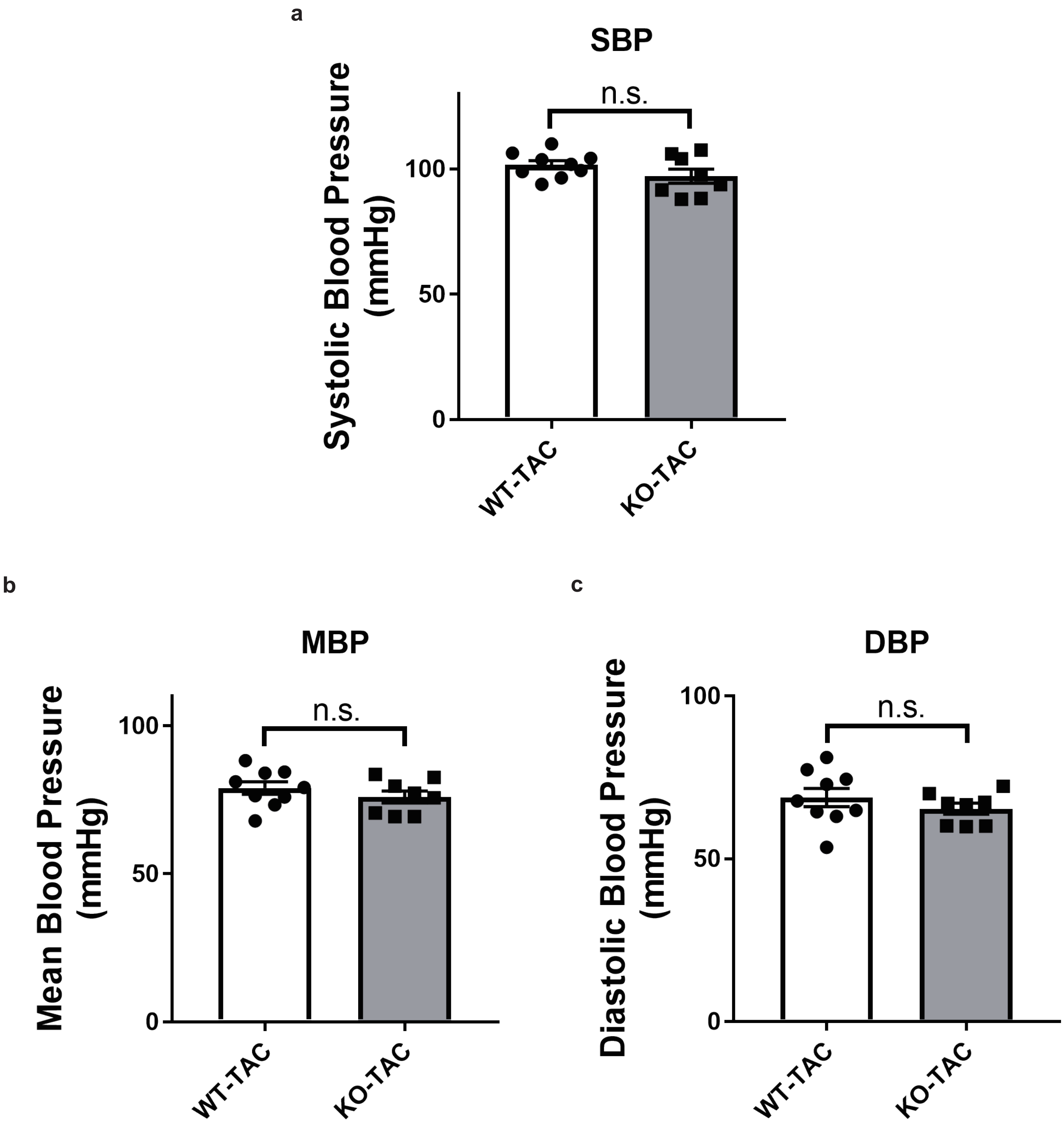

Supplementary Fig. 8. Blood Pressure of *Lionheart*-KO mice subjected to TAC. (**a**) Systolic blood pressure. (**b**) Mean blood pressure. (**c**) Diastolic blood pressure. WT: n = 9; KO: n = 8.

a

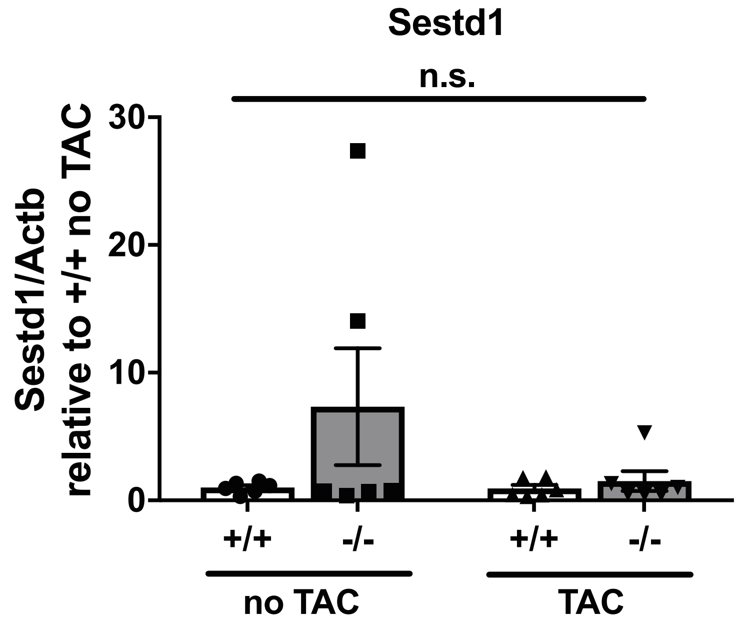

b

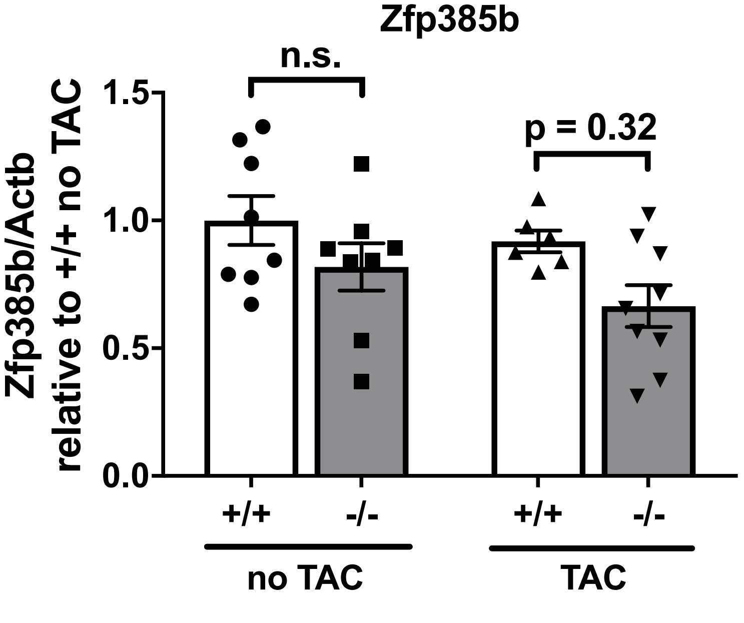

Supplementary Fig. 9. mRNA levels of *Lionheart* neighboring genes in *Lionheart*-KO mouse hearts. (**a**) Sestd1. no TAC: n = 8; TAC: n = 6. (**b**) Zfp385b. no TAC: n = 8; +/+ TAC: n = 6; -/- TAC: n = 9.

a

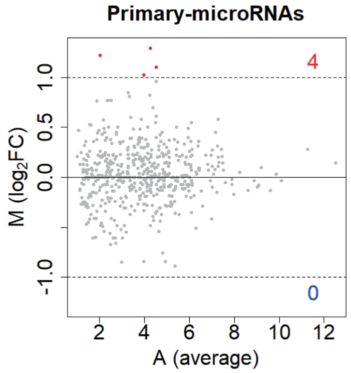

Gene Symbol	Description	Chromosome	Genomic Position	Strand
Mir3962	microRNA 3962	chr1	194822001 - 194822064	+
Mir669a-3	microRNA 669a-3	chr2	10474433 - 10474541	+
Mir1946b	microRNA 1946b	chr9	21613444 - 21613576	-
Mir709	microRNA 709	chr8	84086099 - 84086186	+

b

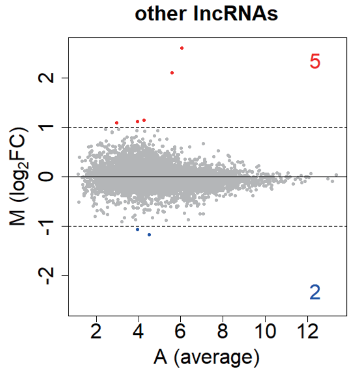

Gene Symbol	Description	Chromosome	Genomic Position	Strand
Gm14443	predicted gene 14443	chr2	175128112 - 177206451	-
2810047C21Rik1	RIKEN cDNA 2810047C21 gene 1	chr7	8086047 - 8093037	-
Slc22a13b-ps	solute carrier family 22 (organic cation transporter), member 13b, pseudogene	chr9	119220491 - 119231692	-
BC023105	cDNA sequence BC023105	chr18	60439295 - 60443848	+
BC023105	cDNA sequence BC023105	chr18	60439295 - 60443848	+

Gene Symbol	Description	Chromosome	Genomic Position	Strand
Snora75; Ncl	small nucleolar RNA, H/ACA box 75; nucleolin	chr1	86351169 - 86351301	-
LOC102634585	uncharacterized LOC102634585	chr1_GL456210_random	157285 - 169279	-

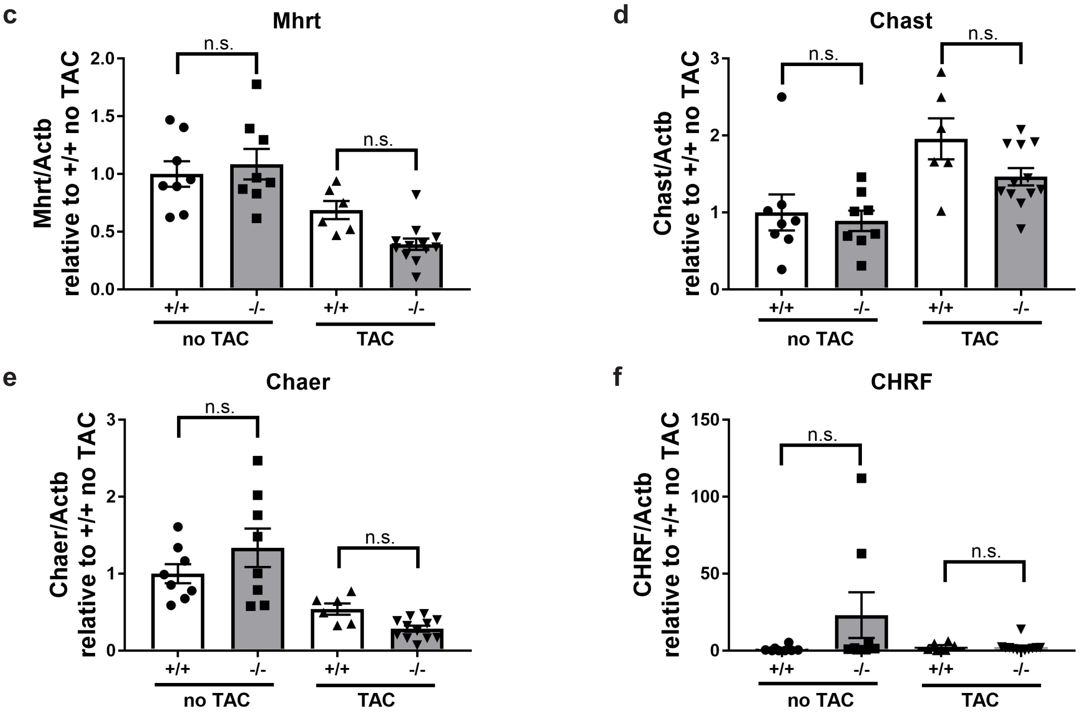

Supplementary Fig. 10. Expression changes of lncRNAs in *Lionheart*-KO mouse hearts after TAC surgery. (**a** and **b**) Results of microarray analyses. Scatter plots of expression levels (x axis) and log2 fold change (y axis). Red dots indicate increased genes (fold change > 2) and blue dots indicate decreased genes (fold change < 0.5). (**a**) Scatter plots of pri-miRNAs and a list of 4 increased pri-miRNAs. (**b**) Scatter plots of other lncRNAs and lists of 5 increased and 2 decreased lncRNAs. (**c**–**f**) Expression levels of cardiac remodeling-associated lncRNAs including Mhrt (**c**), Chast (**d**), Chaer (**e**), and CHRF (**f**). no TAC: n = 8; +/+ TAC: n = 6; -/- TAC n = 12.

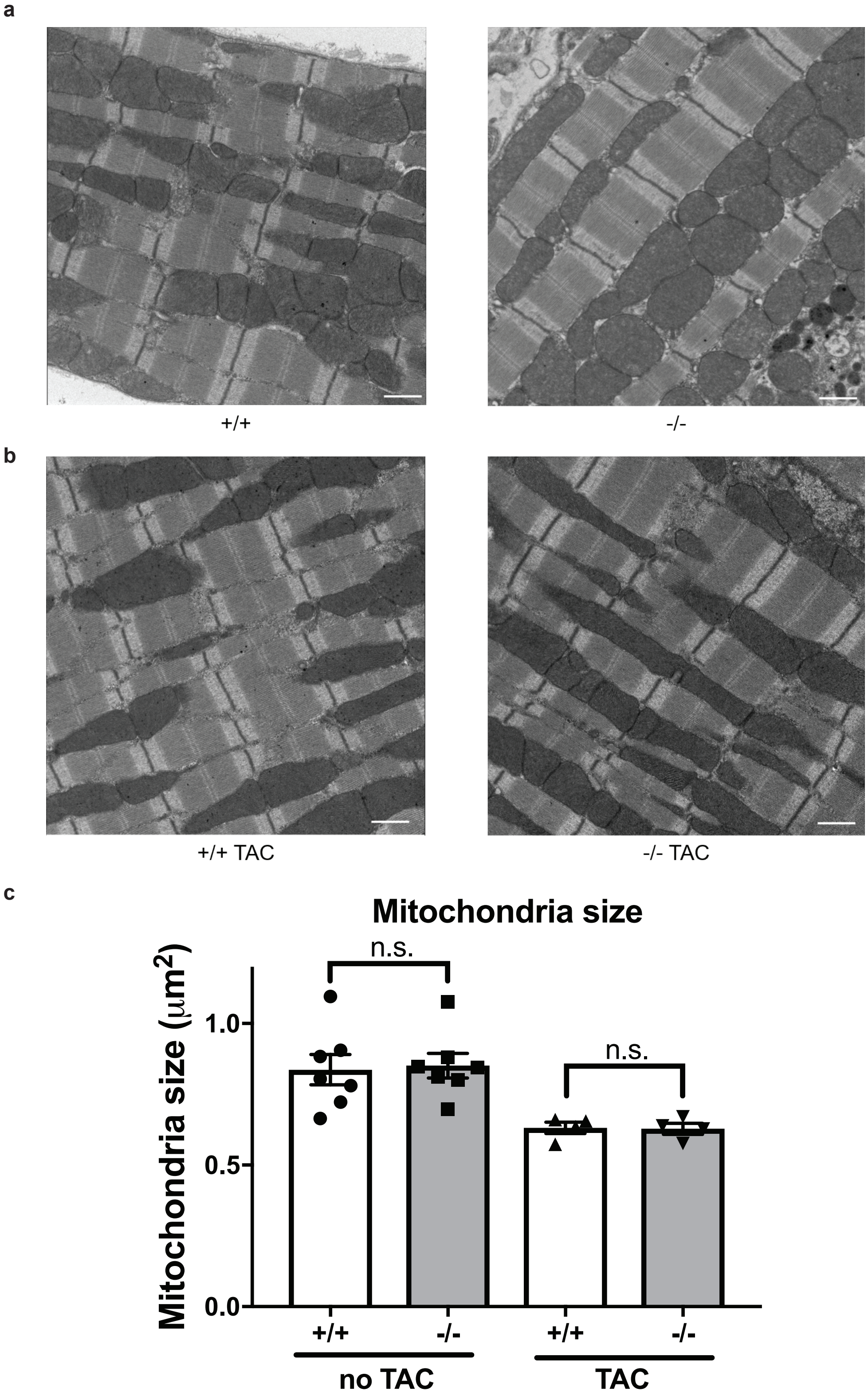

Supplementary Fig. 11. Mitochondrial ultrastructure of *Lionheart*-KO mouse hearts with or without TAC. (**a** and **b**) Representative transmission electron microscopy (TEM) images of hearts from control and *Lionheart*-KO mice. Scale bar = 1 μm. (**a**) Baseline. (**b**) After TAC. (**c**) Quantification of mitochondrial size. no TAC: n = 7; TAC: n = 4.

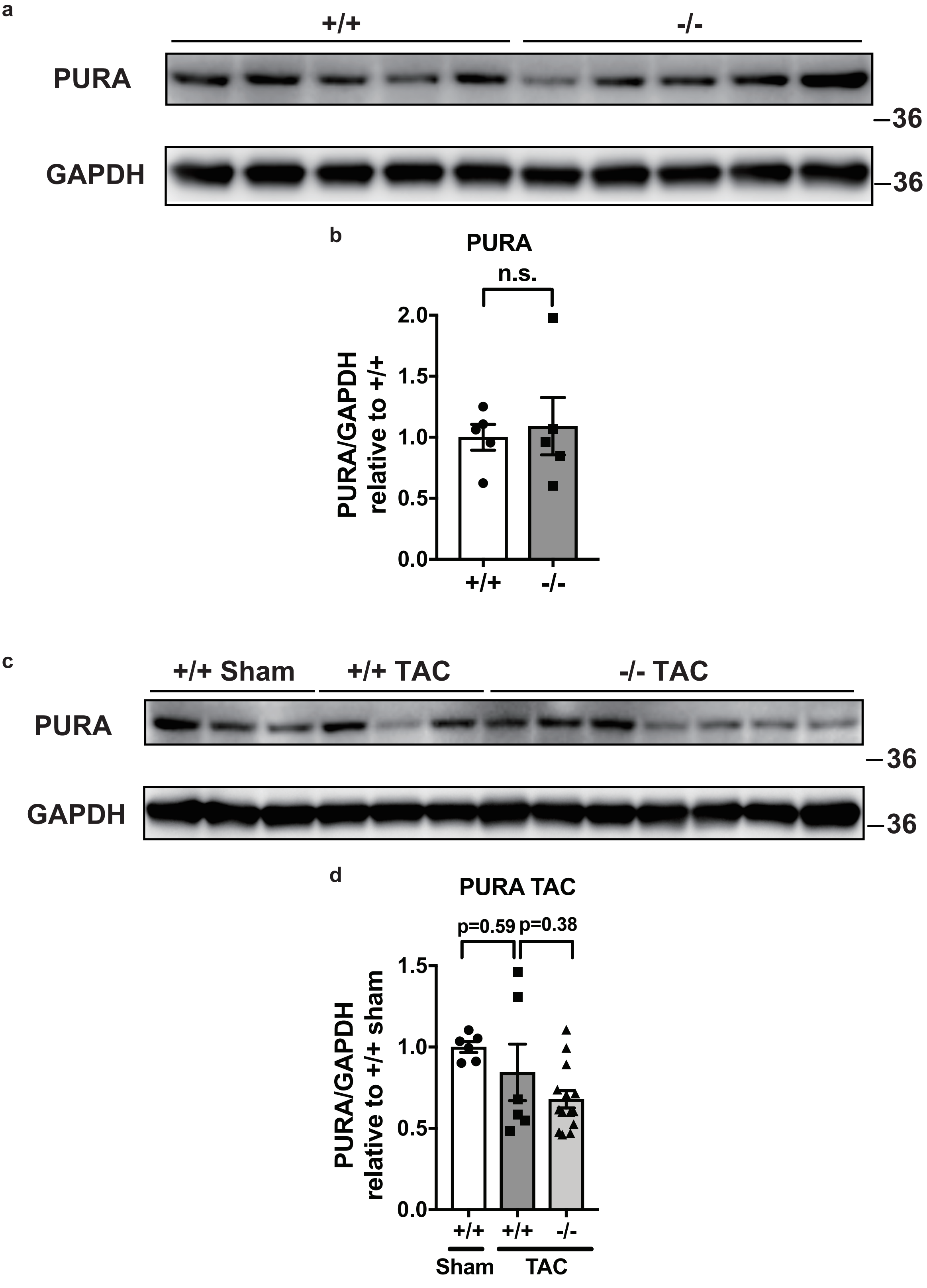

Supplementary Fig. 12. Protein levels of PURA in *Lionheart*-KO mice. (**a** and **b**) PURA levels at baseline. 16 weeks of age. Representative image of western blotting (**a**) and the quantification (**b**). n = 5 in both groups. (**c** and **d**) PURA levels at 8 weeks after TAC. 16 weeks of age. Representative image of western blotting (**c**) and the quantification (**d**). +/+ Sham: n = 6; +/+ TAC: n = 6; -/- TAC: n = 14.

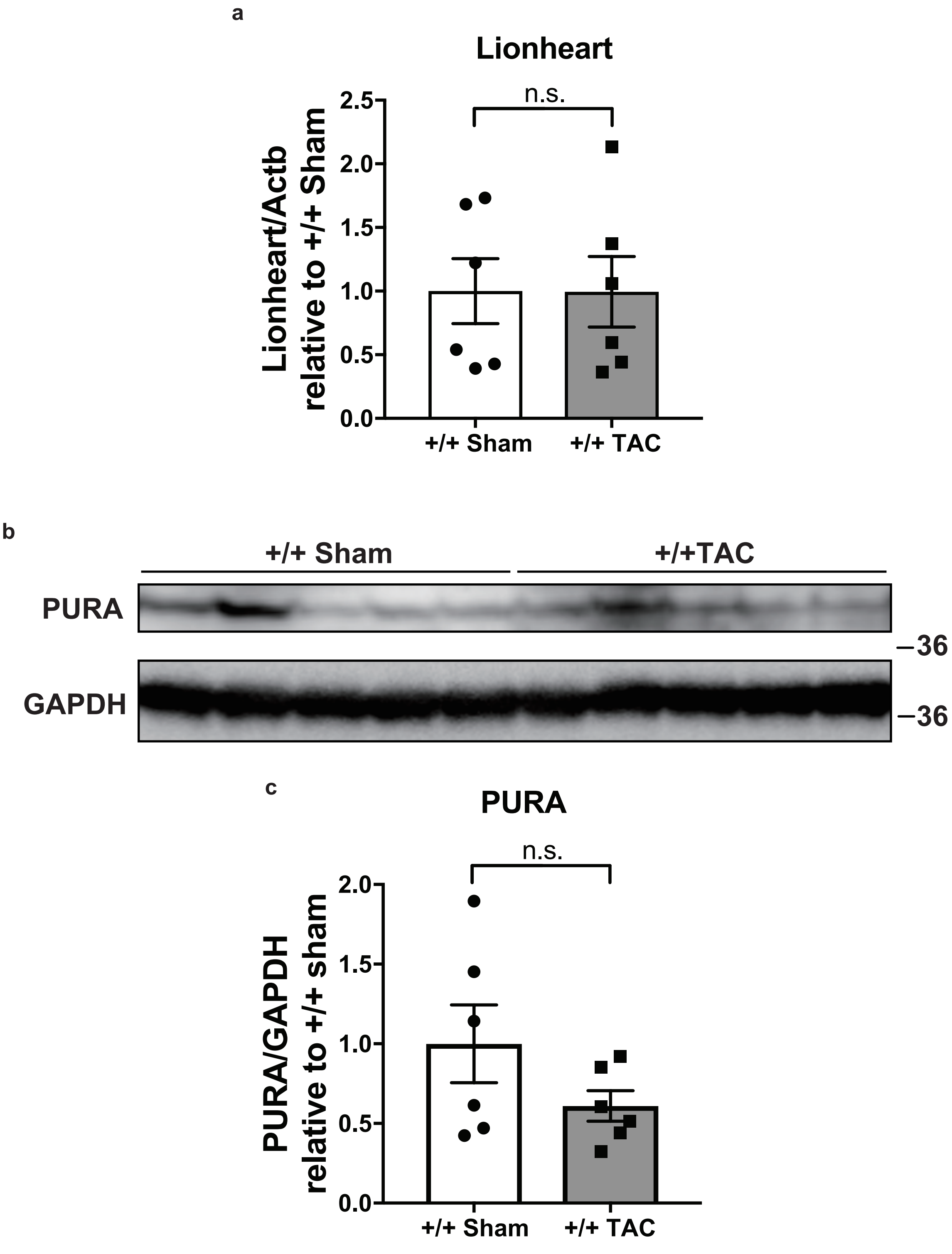

Supplementary Fig. 13. Lionheart levels and PURA protein levels in right ventricles after TAC surgery. (**a**) Lionheart levels. n = 6 in both groups. (**b** and **c**) Representative image of PURA (**b**) and the quantification (**c**). n = 6 in both groups.

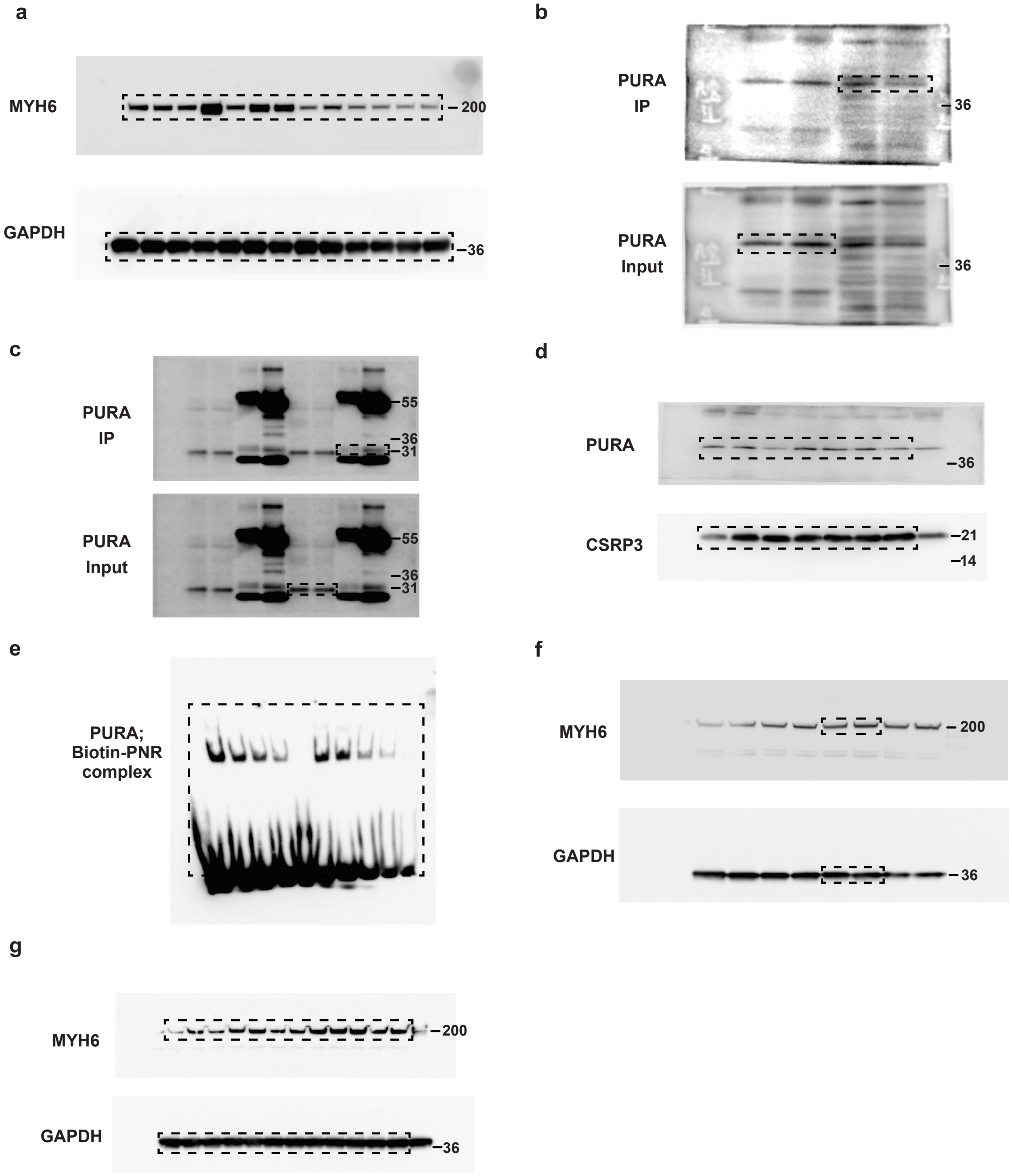

Supplementary Fig. 14. Uncropped immunoblot images in main Figures. (**a**) Fig. 3i. (**b**) Fig. 4b. (**c**) Fig. 4c. (**d**) Fig. 4k. (**e**) Fig. 4m. (**f**) Fig. 4q. (**g**) Fig. 5e.

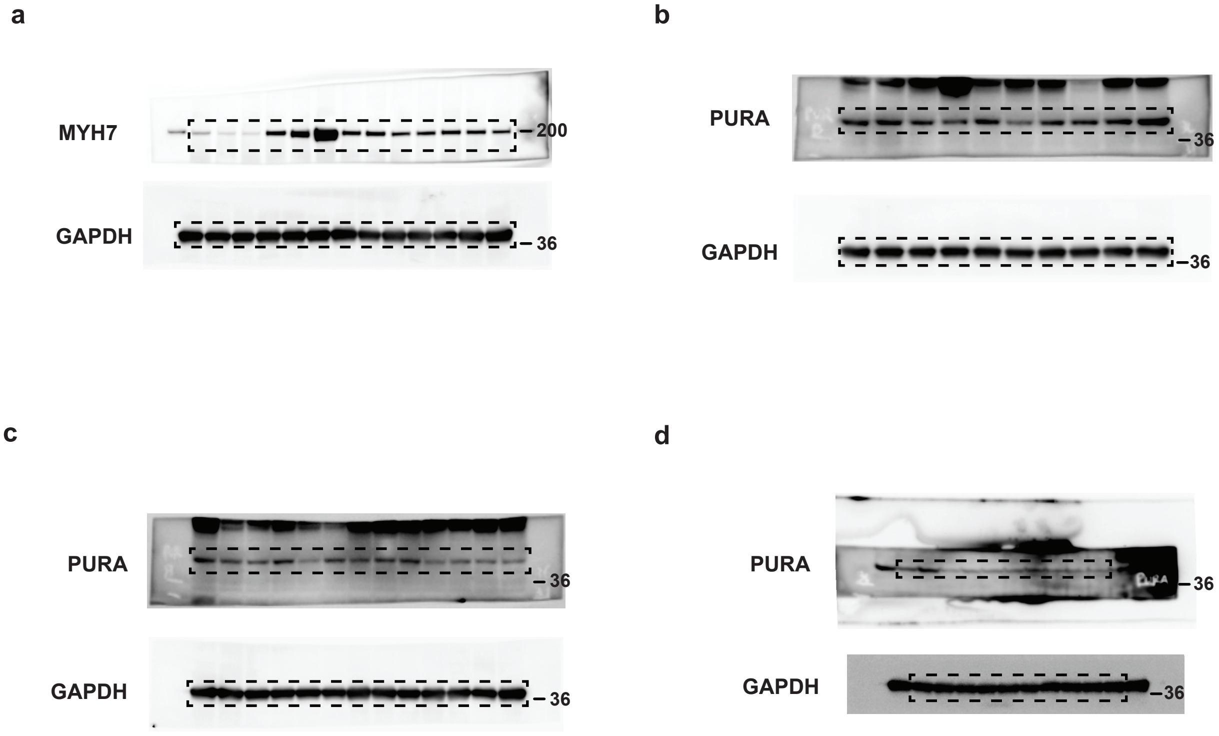

Supplementary Fig. 15. Uncropped western blot images in Supplementary Figures. (**a**) Supplementary Fig. 7f. (**b**) Supplementary Fig. 12a. (**c**) Supplementary Fig. 12c. (**d**) Supplementary Fig. 13b.

Supplementary Table 1. GO-BP term analysis for Lionheart sense-binding protein candidates

	GO-BP Term	P value
1	ATP synthesis coupled proton transport	2.50E-09
2	transport	2.80E-09
3	proton transport	6.20E-09
4	ATP metabolic process	4.70E-08
5	ATP biosynthetic process	2.90E-07
6	tricarboxylic acid cycle	5.00E-05
7	ion transport	8.50E-05
8	metabolic process	1.30E-04
9	translation	4.50E-04
10	fatty acid metabolic process	6.00E-04
11	fatty acid beta-oxidation using acyl-CoA dehydrogenase	1.00E-03
12	regulation of the force of heart contraction	1.50E-03
13	fatty acid beta-oxidation	5.40E-03
14	lipid metabolic process	5.50E-03
15	oxidation-reduction process	6.30E-03
16	cardiac muscle contraction	6.40E-03
17	negative regulation of mitochondrial membrane permeability involved in apoptotic process	7.40E-03
18	leucine catabolic process	9.90E-03
19	succinyl-CoA metabolic process	1.50E-02
20	negative regulation of mitochondrial outer membrane permeabilization involved in apoptotic signaling pathway	1.50E-02
21	mitochondrial acetyl-CoA biosynthetic process from pyruvate	2.00E-02
22	succinate metabolic process	2.50E-02
23	cardiac myofibril assembly	2.90E-02
24	heart contraction	4.10E-02

GO-BP: Gene Ontology-Biological Process

Supplementary Table 2. GO-BP term analysis for Lionheart antisense-binding protein candidates

	GO-BP Term	P value
1	extracellular matrix organization	9.60E-10
2	wound healing	2.00E-06
3	skeletal system development	3.30E-06
4	collagen fibril organization	7.40E-06
5	cellular response to amino acid stimulus	8.90E-06
6	skin development	2.50E-05
7	collagen-activated tyrosine kinase receptor signaling pathway	2.60E-05
8	substrate adhesion-dependent cell spreading	7.10E-04
9	cell adhesion	1.20E-03
10	peptide cross-linking	1.40E-03
11	angiogenesis	1.60E-03
12	blood vessel development	2.20E-03
13	peptide cross-linking via chondroitin 4-sulfate glycosaminoglycan	7.90E-03
14	cartilage development involved in endochondral bone morphogenesis	8.90E-03
15	skin morphogenesis	1.10E-02
16	regulation of embryonic development	1.20E-02
17	protein heterotrimerization	1.90E-02
18	endodermal cell differentiation	2.70E-02
19	endochondral ossification	2.90E-02
20	regulation of cell adhesion	4.10E-02
21	negative regulation of JAK-STAT cascade	4.20E-02

Supplementary Table 3. Patient characteristics in LV biopsy cohort

No.	Diagnosis	Age (years)	Sex	Height (cm)	Body Weight (kg)	BMI (kg/m^2)	mean PCWP (mmHg)	Cardiac Index (L/min)	EF (%)	Cre (mg/dL)	BUN (mg/dL)
1	DCM	33	M	174.1	74.3	24.5	7	2.95	46.2	N/A	N/A
2	DCM	60	M	164.2	70.8	26.3	10	2.26	53.2	0.9	16.9
3	DCM	78	F	158	47.0	18.8	4	2.89	65.2	0.7	20.6
4	DCM	65	F	159	69.5	27.5	5	2.77	38.7	0.8	17.3
5	DCM	34	F	152	50.0	21.6	8	6.62	63	0.6	11.1
6	DCM	60	M	168.9	56.9	19.9	10	2.42	29.5	1.1	20.2
7	DCM, VT	61	F	154	51.0	21.5	4	3.47	52.1	0.5	11.3
8	DCM	40	M	163.8	86.2	32.1	32	1.82	23	1.34	44.9
9	DCM	49	F	153	58.0	24.8	9	2.70	25.2	0.56	18.9
10	DCM	70	F	152	63.6	27.5	14	2.24	23.1	0.98	22
11	Tachycardia-induced CM	60	M	173.5	68.7	22.8	3	1.99	48.4	0.86	17.3
12	DCM	51	M	170.2	63.4	21.9	6	2.43	27.1	0.95	9.1
13	DCM	55	M	157.4	76.7	31.0	7	3.01	43.4	1.17	18.2
14	DCM	56	M	162	62.0	23.6	16	1.84	28.8	0.93	21.4
15	Myocarditis	61	F	157	61.0	24.8	N/A	N/A	41.9	0.59	14.4
16	Myocarditis, AVB	61	F	150	48.0	21.3	N/A	N/A	68.2	0.77	13.7
17	DCM	57	M	175.9	79.3	25.6	24	1.95	20	1.24	25.8
18	DCM	63	M	170.5	78.2	26.9	19	2.07	18.9	1.42	22.9
19	DCM	49	F	146	44.5	20.9	9	3.26	49.3	0.74	11.4
20	DCM	60	F	157.7	64.0	25.7	7	2.62	27	1.04	23.1
21	DCM	42	M	171	93.1	31.8	21	1.96	16.2	1.32	21.8
22	DCM	56	F	154.7	45.2	18.9	5	1.60	20.1	1.01	22.1
23	DCM	56	F	156.5	54.2	22.1	5	2.15	29.2	0.65	11.2
24	DCM, AVB	38	M	175.4	107.4	34.9	N/A	N/A	38.9	9	89.8
25	DCM	63	M	160	57.3	22.4	10	3.64	44.1	7.1	21.9
26	HCM dilated phase	46	M	174	81.2	26.8	8	3.16	30.4	0.87	9.6
27	HCM dilated phase	76	M	163.8	76.3	28.4	19	1.41	39.2	1.81	35.4
28	DCM	26	M	162.2	86.5	32.9	11	3.43	NA	1.15	20.9
29	DCM	71	M	171.6	54.4	18.5	5	4.71	45.2	10.8	62.2
30	DCM	64	M	165	77.3	28.4	9	1.80	31.4	0.93	12.6
31	AVB	46	M	167.8	80.7	28.7	N/A	N/A	N/A	7.86	61.3
32	AVB	64	F	160	62.0	24.2	N/A	N/A	79.4	0.6	111.9
33	DCM	72	M	161	68.0	26.2	23	2.03	33.6	0.87	14.1
34	Myocarditis	44	M	167	61.0	21.9	N/A	N/A	N/A	4.23	52.2
35	DCM	71	M	165.4	59.8	21.9	16	1.73	45.1	1.03	20.2
36	DCM	71	F	165.4	48.8	17.8	20	1.80	12.2	1.13	40.1
37	DCM	49	M	172	71.0	24.0	7	2.26	32.8	0.84	14.3
38	DCM	48	M	165	59.0	21.7	14	2.50	19.9	0.93	14
39	HCM dilated phase	57	F	151	53.0	23.2	11	1.91	34.2	0.73	19.2
40	DCM	50	F	155	48.2	20.1	20	2.06	22.1	1.01	20.1
41	DCM	66	M	163.3	55.5	20.8	25	1.55	29.1	0.83	14.4
42	HOCM	44	M	175	82.0	26.8	13	2.41	51	0.89	14.3
43	Myocarditis	44	F	150	42.0	18.7	18	2.29	N/A	0.63	11.2
44	DCM	60	M	174	60.4	19.9	14	2.32	27	13.56	83.7
45	DCM	55	M	151	56.0	24.6	39	2.36	21.9	0.75	10
46	DCM, Af, MR	44	M	177	73.0	23.3	7	1.68	15.6	1	13
47	DCM	33	M	170	60.2	20.8	27	1.35	19.4	0.96	10.7
48	Myocarditis	30	M	175	60.0	19.6	N/A	N/A	31.8	0.79	20.3
49	Myocarditis	65	F	155	42.0	17.5	N/A	N/A	63.2	0.68	17.5
50	DCM	61	M	168	60.0	21.3	N/A	N/A	19.7	0.98	11.9
51	DCM	72	F	151.4	49.8	21.7	6	3.00	42.8	0.6	12.6

52	DCM	71	M	165.6	47.3	17.3	4	1.43	12.6	1.16	31.7
53	DCM	63	F	153	58.5	25.0	22	1.89	32.8	0.7	15.9
54	Muscular dystrophy, DCM	48	M	162	72.1	27.5	26	1.79	13.3	0.79	17.5
55	DCM	64	F	160	52.9	20.7	21	1.96	28	0.65	19.2
56	DCM, LBBB	73	F	145	37.2	17.7	10	2.41	17.8	0.54	12.4
57	DCM	64	M	179	73.3	22.8	5	1.82	26.3	0.77	18.6
58	HCM dilated phase	65	M	160.6	52.8	20.5	19	1.72	31.2	0.91	14.4
Mean		56.1		162.7	63.0	23.6	13.3	2.40	34.3	1.72	24.3

BMI: Body mass index, PCWP: Pulmonary capillary wedge pressure, EF: Ejection fraction (%), Cre: Creatinine (mg/dL), BUN: Blood urea nitrogen (mg/dL), DCM: Dilated cardiomyopathy, M: Male, F: Female, VT, Ventricular tachycardia, CM: Cardiomyopathy, AVB: Atrioventricular block, HCM: Hypertrophic cardiomyopathy, HOCM: Hypertrophic obstructive cardiomyopathy, Af: Atrial fibrillation, MR: Mitral regurgitation, LBBB: Left bundle branch block

Supplementary Table 3 (continued). Patient characteristics in LV biopsy cohort

No.	Diagnosis	eGFR (mL/ min)	Hb (g/dL)	RBC ($x10^4$/ μL)	WBC ($x10^2$/ μL)	BNP (pg/ mL)	HbA1c (%)	T-Cho (mg/dL)	TG (mg/dL)	HDL-C (mg/dL)	LDL-C (mg/dL)
1	DCM	N/A	13.8	465	62.2	6.5	5	204	325	49	111
2	DCM	67.2	13	443	77.2	153	4.6	145	103	31	83
3	DCM	60.7	11.6	438	52.3	40.4	5.7	157	29	58	80
4	DCM	55.2	13.9	446	70.4	57.1	5.4	214	131	46	133
5	DCM	91.1	12.9	449	82.4	12.1	5.3	175	146	63	92
6	DCM	54	14	495	58.6	978	4.9	216	129	58	130
7	DCM, VT	94.1	12.5	411	69.4	N/A	4.6	235	154	61	150
8	DCM	48.9	14.8	515	57.4	1040	6.6	155	71	27	109
9	DCM	88.5	12	387	144.3	1090	5.5	96	64	19	69
10	DCM	43.3	14	455	63.1	455	5.3	134	31	49	71
11	Tachycardia-induced CM	68.1	17.6	542	68.8	302	5.1	173	149	43	111
12	DCM	66.4	15	477	83.7	455	5.8	N/A	102	45	123
13	DCM	51.7	14.3	468	58.3	20.6	5.7	235	261	65	138
14	DCM	66.2	15	480	42.1	163	4.8	169	64	45	107
15	Myocarditis	78.1	13.9	442	51.9	1320	5.5	182	40	34	129
16	Myocarditis, AVB	58.6	14	438	56.7	45.6	5.1	224	191	33	145
17	DCM	48	17.1	576	67.7	762.9	5.3	195	110	57	116
18	DCM	40.4	14.8	489	63	1211.7	6.3	153	130	51	86
19	DCM	65.2	11.7	444	83.9	4.4	4.6	341	198	71	240
20	DCM	42.4	12.4	405	39.6	193.4	5.1	183	102	51	98
21	DCM	49	18.9	663	92.9	222.5	5.7	219	174	42	139
22	DCM	44.7	14.7	486	74.7	2662.5	5.8	168	109	43	106
23	DCM	97.9	12	396	43.4	900.5	7.9	123	49	30	80
24	DCM, AVB	6.2	8.3	261	90	484.4	5.2	N/A	N/A	N/A	N/A
25	DCM	6.9	9.4	321	27.1	1004.2	N/A	208.6	88	47	144
26	HCM dilated phase	75.3	13.9	464	109.5	74.9	6.3	272	194	44	182
27	HCM dilated phase	29.2	16.9	544	61.5	521.5	5.9	165	184	41	86
28	DCM	65.4	20.1	613	105.1	58.1	5.7	317	257	51	229
29	DCM	4.2	9.6	284	29.2	371	4.4	189	32	57	107
30	DCM	63.7	13.2	535	67.1	245.7	7	146	165	35	94
31	AVB	6.7	10.8	392	113.7	N/A	7.2	N/A	101	44	80
32	AVB	76	14.6	492	85.3	1075.8	5.2	318	36	85	210
33	DCM	66.2	15.1	504	56	574	5.4	145	144	31	93
34	Myocarditis	13.5	13	419	108.7	288.3	5.5	149	82	53	69
35	DCM	55.3	15.4	514	50.6	251.9	6.3	175	88	41	116
36	DCM	49.9	13.7	430	54.3	1309.4	7.1	185	98	61	104
37	DCM	77.3	16.4	489	61.9	208.5	5.4	260	63	41	155
38	DCM	69.6	13.6	461	68	1138.3	6.4	N/A	76	67	111
39	HCM dilated phase	63.4	11.8	386	54.6	188.2	9.8	215	240	42	136
40	DCM	46.1	15.5	514	62.2	697	5.5	181	71	71	96
41	DCM	71.5	16.6	517	84.6	1563.7	7	163	81	35	112
42	HOCM	74.9	15.2	474	47.4	224.1	5.3	N/A	164	32	121
43	Myocarditis	80.8	13.9	455	123.2	847.3	6.1	121	68	37	67
44	DCM	3.5	11.7	368	75.2	4969.4	5.7	177	244	43	85
45	DCM	84.1	12.9	487	70.1	979.8	5.5	N/A	63	50	143
46	DCM, Af, MR	65.5	15.6	485	58.6	95.9	5.2	168	131	34	108
47	DCM	74.4	15.1	515	80.4	1099.7	5.7	222	95	62	141
48	Myocarditis	94.6	14.8	468	96.2	763.3	5.8	N/A	97	44	76
49	Myocarditis	63.3	13.6	416	42.2	22	5.3	N/A	88	63	107
50	DCM	61	14.4	429	66.2	288.2	5.5	222	95	62	141
51	DCM	73.5	13.6	441	42.1	291.9	5.3	180	50	65	105

52	DCM	48.5	15.7	487	41	923.7	6	170	53	37	122
53	DCM	64.5	16.7	479	47.5	344.5	7.8	197	99	67	110
54	Muscular dystrophy, DCM	82.7	14.1	445	44.9	434.9	5.7	221	171	67	120
55	DCM	69.6	11.2	428	58	1239.4	5.8	151	63	39	99
56	DCM, LBBB	82.1	11.4	383	35.5	1320.8	5.4	218	170	43	141
57	DCM	78.3	13.6	438	53.4	37.8	5.8	133	111	34	77
58	HCM dilated phase	64.9	14.4	437	48.6	258.9	6	208	192	55	125
Mean		59.5	14.0	458.4	67.0	648.1	5.8	191.4	119.6	48.3	116.8

eGFR: estimated glomerular filtration rate, Hb: Hemoglobin, RBC: Red blood cell, WBC: White blood cell, BNP: Brain natriuretic peptide, T-Cho: Total cholesterol, TG: Triglyceride, HDL-C: High-density lipoprotein cholesterol, LDL-C: Low-density lipoprotein cholesterol
